# Von Hippel–Lindau/Hypoxia Inducible Factor Axis in Glioblastoma

**DOI:** 10.3390/ijms26209979

**Published:** 2025-10-14

**Authors:** Itamar Flores, Aleli Salazar, Verónica Pérez de la Cruz, Tamara Mena-Guerrero, Javier Angel Navarro Cossio, Rubén Figueroa, Mario Eugenio Cancino-Diaz, Benjamin Pineda

**Affiliations:** 1 Neuroimmunology and Neuro-Oncology Unit, National Institute of Neurology and Neurosurgery “Manuel Velasco Suárez”, Mexico City 14269, Mexico; itamar.flores@innn.edu.mx (I.F.); aleli.salazar@innn.edu.mx (A.S.); tamara.menagu@gmail.com (T.M.-G.); jnavarroc2302@alumno.ipn.mx (J.A.N.C.); rubenfr@ciencias.unam.mx (R.F.); 2 Neurobiochemistry and Behavior Laboratory, National Institute of Neurology and Neurosurgery “Manuel Velasco Suárez”, Mexico City 14269, Mexico; veped@yahoo.com.mx; 3 Laboratorio de Inmunorregulación, Departamento de Inmunología, Escuela Nacional de Ciencias Biológicas, Instituto Politécnico Nacional, Mexico City 11340, Mexico; 4 Laboratorio de Inmunología Aplicada, Departamento de Inmunología, Escuela Nacional de Ciencias Biológicas, Instituto Politécnico Nacional, Mexico City 11340, Mexico

**Keywords:** hypoxia, gliomas, pVHL, oncogenic signaling

## Abstract

Glioblastoma (GBM) is the most aggressive and lethal primary brain tumor, characterized by rapid proliferation, extensive vascularization, and resistance to conventional therapies. A feature of the GBM microenvironment is hypoxia, which activates a wide range of adaptive responses orchestrated mainly by the hypoxia-inducible factor (HIF). The Von Hippel–Lindau protein (pVHL) is a central regulator of HIF stability, inducing proteasomal degradation under physiological conditions. However, in GBM, the pVHL is frequently mutated or functionally inactivated by several mechanisms, including microRNA regulation, post-translational modifications, or degradation by specific E3 ubiquitin ligases. This loss of function results in persistent HIF activation, thereby enhancing the oncogenic and pro-angiogenic environment that contributes to the progression and aggressiveness of GBM. This review focuses on the multifaceted roles of the pVHL-HIF axis and proposes it as a key driver of GBM malignancy.

## 1. Introduction

Glioblastoma Multiforme (GBM) is the most common and aggressive malignant primary brain tumor. Originating from glial cells, GBM is characterized by marked heterogeneity, invasiveness, and a high mortality rate, primarily affecting adults between 40 and 65 years of age, with an incidence rate of 3.23 cases per 100,000 population [1]. Current standard therapy consists of maximal surgical resection, followed by fractionated radiotherapy (total dose of 60 Gy) and chemotherapy with Temozolomide (TMZ), which is the first-line chemotherapeutic agent. Despite this multimodal treatment, the life expectancy for patients with GBM remains approximately 12–15 months, and the five-year survival rate is below 5% [2,3,4].

GBM presents a high proliferation rate and displays dissimilar oxygen concentrations across the tumor mass. These conditions contribute to the development of edema and necrotic zones, particularly within the tumor core, where the oxygen rate is critically low. These pathological characteristics are used for the diagnosis of GBM [5].

Among the hallmarks of cancer, hypoxia and angiogenesis are especially prominent in GBM, contributing to its progression and aggressiveness. These processes primarily involve the hypoxia-inducible factor (HIF), which shows high expression in tumor masses and glioma cell lines [6]. The principal negative regulator of HIF activity is the Von Hippel–Lindau protein (pVHL), which mediates HIF degradation under normoxic conditions. Studies have linked alterations in pVHL expression, inhibition, or degradation to poor prognosis in various cancers, such as clear cell renal cell carcinoma [7]. More recently, pVHL deregulation has also been implicated in the pathogenesis of CNS tumors [8].

This review focuses on understanding the VHL/HIF signaling axis in GBM, its implications in tumor development, and its potential as a therapeutic target for the treatment of brain tumors.

## 2. Hypoxia in GBM: Contributing Factors and Tumor Microenvironment Interactions

**Figure 1:**
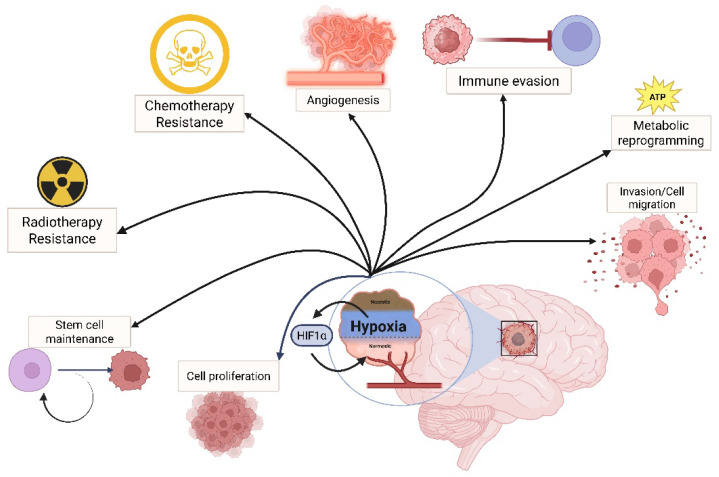
HIF as a regulator of cancer hallmarks in GBM. In a highly hypoxic environment, such as in GBM, the HIF complex regulates the expression of genes involved in tumoral progression, such as cell proliferation, migration, stemness maintenance, therapy resistance, angiogenesis, metabolic adaptation, and immune evasion.

Hypoxia is a condition characterized by reduced or insufficient oxygen availability. In the healthy brain, oxygen concentrations range from approximately 2.5% to 12.5%. While transient hypoxia in normal tissues plays an essential role in regulating processes such as cell proliferation, metabolism, differentiation, and the activity of microglia and astrocytes, which modulates immune response [9], sustained or chronic hypoxia disrupts cellular homeostasis; its disruption may lead to tissue damage, necrosis, and loss of tissue function or viability. Importantly, hypoxia has been considered a critical factor in cancer progression, particularly in solid tumors [10] (Figure 1).

In GBM, oxygen levels are significantly reduced, often falling between 0.1% and 2.5% [11]. The hypoxic state is primarily a consequence of high rates of cellular proliferation and rapid tumor growth, which significantly reduces oxygen availability within the tumor microenvironment (TME), especially in regions far from arterial blood supply. These hypoxic conditions contribute to the formation of necrotic zones surrounded by pseudopalisading cell clusters (histopathological hallmark of GBM) [12].

Multiple factors contribute to hypoxia in GBM. Microthrombosis is a key contributor, as it impairs oxygen delivery, induces a hypercoagulable state, and promotes necrosis, collectively driving tumoral progression [13]. GBM patients frequently develop thrombotic complications such as venous thromboembolism, with an incidence ranging from 24 to 30% [14]. Additional contributors to tumor hypoxia include elevated metabolic demands, structurally and functionally abnormal vasculature, and impaired oxygen diffusion due to the high cellular density of the tumor mass [1].

GBM is a highly heterogeneous tumor, and hypoxic niches play a role in tumor development. Spatial transcriptomics and single-cell analysis have confirmed the differential presentation of the Warburg effect in the hypoxic zone, while the peritumoral zones exhibit glutamine metabolism and fatty acid oxidation [15,16]. The distribution of cells and their multilayer organization correlate with the distance to the hypoxic/necrotic region, as reported by Greenwald et al. (2024) with their spatial transcriptomics/proteomic approach [17]. Recent studies have also revealed the preference of glioma cells in hypoxic conditions to adopt a mesenchymal-like cell state, which is highly invasive and promotes immune suppression. Hypoxia drives shared and distinct transcriptomic changes in two invasive glioma stem cell lines. Hypoxia is also a driver in PD-L1 expression, in combination with the co-expression of PD-L1 and HIF-1α in glioma cells under hypoxia [18,19,20].

**Figure 2:**
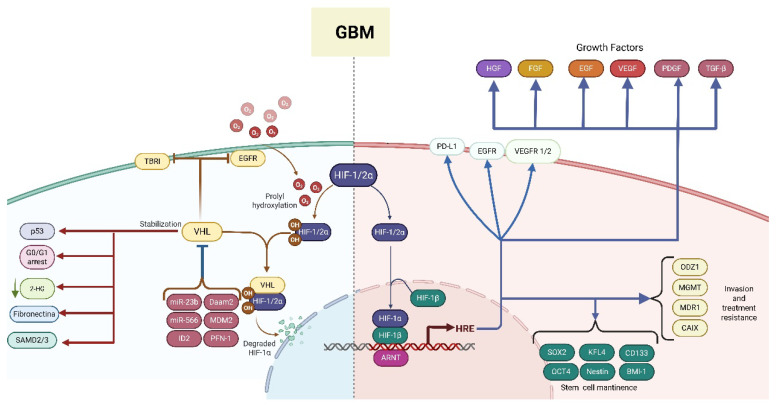
HIF regulation is aberrant in glioblastoma (GBM). In GBM, VHL expression is downregulated, whereas the expression of negative regulators of VHL is upregulated, leading to deficient regulation of critical processes, including cell cycle regulation, which promotes the expression of growth factor-induced proliferation and the degradation of HIF1/2 α (left side). As a result, the HIF signaling cascade downstream becomes activated, and the HIF complex binds to hypoxia-response elements (HREs) in the untranslated regions of multiple target genes, promoting their transcription. These molecular events collectively support cell growth and proliferation, stemness maintenance, invasion, and treatment resistance (right side). The red lines highlight VHL functions that become impaired in GBM. Blue lines show signaling pathways and biological processes that are activated/stimulated in GBM. Solid arrows indicate a positive regulation, while bar-headed arrows indicate a negative regulation. The abbreviations correspond to receptors and growth factor: EGFR (epidermal growth factor receptor), TBRI (transforming growth factor beta receptor), VEGFR 1/2 (vascular endothelial growth factor receptor 1/2), PD-L1 (programmed cell death ligand 1), HGF (hepatocyte growth factor), FGF (fibroblast growth factor), EGF (epidermal growth factor), VEGF (vascular endothelial growth factor), PDGF (platelet-derived growth factor), TGF-β (transforming growth factor β); signaling cascade mediator and gene transcription regulators: HIF (hypoxia-inducible factor), VHL (Von Hippel–Lindau), p53, SMAD2/3 (SMAD Family Member 2/3), miR-566, miR-23b, MDM2 (mouse double minute 2 homolog), Daam2 (Dishevelled-associated activator of morphogenesis 2), ID2 (inhibitor of DNA binding 2), ARNT (aryl hydrocarbon receptor nuclear translocator), HREs, SOX2 (sex-determining region Y-box 2), OCT4 (Octamer-binding transcription factor 4), KFL4 (Kruppel-like factor 4), BMI-1 (B-cell-specific Moloney murine leukemia virus integration site 1); enzymes, transporters, and more: MGMT (O6-methylguanine-DNA methyltransferase), CAIX (carbonic anhydrase IX), MDR1 (multidrug resistance 1), ODZ1 (also known as Teneurin-1), PFN-1 (profilin-1), CD133 (also known as Prominin-1); and metabolites: 2-HG (2-hydroxyglutarate).

Beyond its role in tumor biology, hypoxia contributes to resistance against radiotherapy and chemotherapy. In this context, hypoxia induces the expression of resistance genes, such as the multidrug resistance 1 gene (MDR1) [21]. This pathway plays a pivotal role in mediating chemoresistance to GBM therapy. Additionally, hypoxia also modulates the expression of O (6)-methylguanine-DNA methyltransferase (MGMT), a key DNA repair enzyme implicated in resistance to TMZ. Notably, CD133+ glioma stem cells cultured under hypoxic conditions exhibit increased MGMT expression, which contributes to TMZ resistance [22,23,24] (Figure 2, right side).

One of the key consequences of hypoxia is that it induces a stem cell-like phenotype in non-tumoral cells by upregulating several stem cell markers, including CD133 (which drives tumor metastasis, drug resistance, and recurrence), OCT4 (a POU family member that maintains the undifferentiated state of embryonic stem cells and promotes their proliferation), BMI-1 (which preserves stem cell properties by preventing premature senescence), Nestin (an intermediate filament protein associated with self-renewal capacity), and SOX2 (a transcription factor that acts synergistically with OCT4 to regulate and maintain cellular pluripotency). Additionally, both in vivo and in vitro studies have demonstrated that hypoxia induces CD133 glycosylation, which prevents its degradation and supports its expression at the cell membrane; therefore, it aids the establishment and maintenance of glioma stem cells (GSCs) (Figure 2, right side). Also, hypoxia stimulates cellular plasticity by acidifying the TME and stabilizes the HIF [25,26,27,28].

Necrotic and pseudopalisading zones within GBM promote the expression of GPR133 (ADGRD1), a member of the adhesion G protein–coupled receptor (AGPCR) family, specifically in CD133+ glioma stem cells. This process is HIF1α-dependent; it has been demonstrated both in vivo and in vitro. The activation of this signaling pathway leads to increased cyclic adenosine monophosphate (cAMP) levels, resulting in cellular proliferation and tumor growth [29].

Hypoxia also induces a metabolic shift from oxidative phosphorylation to glycolysis by upregulating carbonic anhydrases, lactate transporters, and H^+^/HCO3^−^ exchangers. These changes exacerbate TME acidification, which in turn stabilizes heat shock protein 90 (HSP90), supporting HIF activity and promoting tumor cell proliferation in GBM cell lines such as G55, G121, G141, and G142 [30,31].

Interestingly, Pistollato et al. conducted a study using adult brain tumor tissues from patients with GBM and demonstrated that CD133+/MGMT+ GBM stem cells localize predominantly to the inner core of the tumor mass. The authors proposed that these cells confer resistance to TMZ through MGMT upregulation driven by the hypoxic microenvironment, in contrast to the cells located in the more vascularized peripheral regions [32].

### Angiogenesis: A Key Hypoxia-Driven Mechanism in GBM

Angiogenesis occurs when new blood vessels develop from pre-existing vasculature. Pro-angiogenic and anti-angiogenic factors regulate it. It participates in several biological processes, such as embryonic development, tissue repair, and menstruation, among others [33]. In cancer, however, angiogenesis supports tumor growth by supplying oxygen and nutrients through the formation of new vessels, vascular mimicry, and vessel co-option [34].

In GBM, the hypoxic microenvironment strongly promotes angiogenesis [35]. Hypoxia stabilizes HIFs, which in turn upregulate the expression of pro-angiogenic factors, such as vascular endothelial growth factor (VEGF) [36,37,38]. VEGF, an endothelial-specific mitogen, binds to its receptor (VEGFR), promoting receptor dimerization and autophosphorylation, initiating downstream signaling cascades that promote endothelial cell proliferation, migration, and new vessel formation [39]. VEGF and its receptors (VEGFR-1 and VEGFR-2) are overexpressed in GBM and regulate genes involved in cell progression, survival, vascular permeability, and invasion [40,41]. Although hypoxia primarily drives VEGF expression, studies have shown that VEGF mRNA levels can also increase under normoxic conditions in high-cell-density U87 cultures [42].

Despite the robust angiogenic response, the vasculature in GBM is abnormal, characterized by immature vessels, nonfunctional, tortuous, and thickened membranes. This abnormal vasculature is inefficient in reversing the hypoxic state, further exacerbating tumor progression [43,44].

In addition to VEGF, several angiogenic receptors and factors are upregulated in GBM, stimulating angiogenesis through the activation of oncogenes and/or downregulation of tumor suppressor genes. These include transforming growth factor-beta (TGF-β), hepatocyte growth factor (HGF), fibroblast growth factor (FGF), angiopoietin-1, and epidermal growth factor (EGF) [45] (Figure 2, right side).

Given its central role in GBM vascularization, the VEGF signaling pathway has been a primary therapeutic target [34]. Bevacizumab, an anti-VEGF therapy, is the only currently approved treatment for GBM. While it improves progression-free survival, its effects on overall survival remain marginal [46,47,48,49].

Beyond classical angiogenesis, GBM tumors also exhibit vasculogenic mimicry, a phenomenon in which GSCs form perfusable endothelial-like channels independent from endothelial cells. CD133+ GSCs form blood vessels de novo, reinforcing the notion that GSCs contribute to both tumor maintenance and neovascularization [50]. Moreover, VEGF also promotes the proliferation of GSCs. VEGF binds to VEGFR2 on GSC membranes, stimulating cell proliferation [51].

## 3. The Role of HIF in GBM

A primary regulator of cellular response to hypoxia is the HIF complex, a transcription factor composed of α and β subunits. Both subunits share basic helix-loop-helix and PAS domains. Under normoxia, oxygen-dependent mechanisms keep the α subunit in the cytoplasm, where it undergoes proteasomal degradation. In contrast, under hypoxic conditions, the α subunit is stabilized, translocated to the nucleus, and heterodimerizes with the β subunit, also known as the aryl hydrocarbon receptor nuclear translocator (ARNT), expressed and located in the nucleus [52].

Once stabilized, the HIF complex binds to specific DNA regions, functioning as a transcription factor for hypoxia-responsive genes that regulate processes such as metabolic reprogramming, proliferation, motility, stem cell maintenance, and expression of angiogenic factors like vascular endothelial growth factor (VEGF), erythropoietin (EPO), and platelet-derived growth factor (PDGF), all of which contribute to progression [6,53].

There are three isoforms of HIF-α, HIF-1α, HIF-2α, and HIF-3α, of which HIF-1α and HIF-2α play dominant roles in oxygen sensing and cellular adaptation to hypoxic conditions. Their expression follows a temporal pattern: HIF-1α is rapidly induced during early hypoxia but declines under prolonged hypoxic conditions (at 1% O_2_), whereas HIF-2α expression increases progressively during chronic hypoxia. Researchers have observed this expression shift in several types of cancer, including neuroblastoma, breast cancer, prostate cancer, and renal cell carcinoma [54].

Recent bioinformatic analysis of RNA-seq data from The Cancer Genome Atlas (TCGA) and the Chinese Glioma Genome Atlas (CGGA) database revealed that both HIF-1α and HIF-2α are significantly overexpressed in GBM tumors as compared to normal brain tissue [55]. Moreover, elevated expression of HIF-1α has been correlated with poor prognosis and higher tumor grade in gliomas [56]. HIF-1α also regulates iron metabolism by directly binding to the hypoxia response element 2 (HRE-2) in the promoter region of the ferritin light chain (FTL), upregulating its expression. Notably, FTL expression is significantly higher in high-grade gliomas compared to low-grade gliomas. Additionally, it has been shown that FTL knockdown inhibits glioma cell proliferation and increases apoptosis in cells treated with TMZ, suggesting that FTL may contribute to TMZ resistance in glioma, and its regulation by HIF-1α represents a potential therapeutic target [57].

HIF-1α also influences immune escape mechanisms in GBM. Under hypoxic conditions, it enhances the expression of programmed cell death ligand-1 (PD-L1). Pharmacological inhibition of HIF-1α decreases PD-L1 expression. In the murine glioma model, the combination of HIF-1α inhibitor with anti-PD-L1 antibodies induces a synergistic reduction in tumor growth compared with the treatments alone [19]. The TME in GBM is predominantly immunosuppressive. Additionally, the HIF restricts mitochondrial glucose oxidation in tumor-infiltrating T-reg cells under hypoxic conditions, promoting their migration towards the tumor site [58].

HIF-1α is a central driver of tumor metabolic reprogramming, orchestrating the shift from mitochondrial oxidative phosphorylation to aerobic glycolysis. It upregulates key metabolic enzymes and transporters, including carbonic anhydrase IX (CAIX) (a key pH regulator in cancer cells), promoting migration in U87 and U251 glioma cells [59]. Additionally, HIF-1α positively modulates the expression of the proline 4-hydroxylase α subunit (P4HA1), leading to the production of oncometabolites such as succinate, which in turn inhibits PGK1 degradation, favoring aerobic glycolysis [60].

HIF-2α is strongly associated with brain tumors, including neuroblastoma and GBM, under intense and chronic hypoxic conditions [61]. HIF-2α is more prominently expressed in chronic hypoxia and plays a role in the maintenance of cancer stem cells (CSCs). HIF-2α promotes the expression of Nestin and CD133, markers of glioma stemness [62,63]. It also regulates the transcription of teneurin transmembrane protein 1 (ODZ1), a molecule involved in cytoskeletal remodeling and invasion [64]. Moreover, HIF-2α enhances the expression of class III β-tubulin (tubulin beta-3 chain), a protein associated with tumor growth and chemotherapy resistance in GL15 and U87 GBM cell lines [62].

Moreover, HIF activity also impacts epidermal growth factor receptor (EGFR) signaling, a key driver of tumor progression in GBM, where each tumor cell expresses more than 1 × 10^6^ EGFR molecules, which is higher than the physiological contents [65]. EGFR is overexpressed and mutated in 25–33% of cases, with the EGFRvIII variant being the most common mutation associated with poor clinical prognosis [66].

In hypoxic conditions, HIF-1α and HIF-2α promote the expression of EGFR and its ligand, epithelial growth factor (EGF), creating a feedback loop that amplifies EGFR signaling [67]. Additionally, HIF-2α induces transforming growth factor-alpha (TGFα) expression and enhances EGFR mRNA translation, increasing both its stability and activity [68]. EGFR activation upregulates the expression of reticulocalbin 1 (RCN1), which contributes to cell survival by inhibiting apoptosis, reducing the expression of the anti-apoptotic protein Bcl-2 and decreasing caspase 3/7 activity [69].

HIF-1α and HIF-2α synergistically regulate the malignant progression of GBM. In this study, simultaneous knockout of both isoforms led to a significant reduction in the experimental tumor growth, size, and weight, resulting in prolonged survival. Furthermore, pathway analysis showed that the deletion of each isoform affected biological processes: HIF-1α knockout disrupted cellular metabolic pathways, whereas HIF-2α knockout impacted cell invasion-related pathways [55]. Dual knockout of both HIF-1α and HIF-2α affected gene expression programs involved in cell cycle regulation, apoptosis, and cellular metabolism. Further studies using GBM patient-derived cells have corroborated that simultaneous inhibition of HIF-1α and HIF-2α impacts cell proliferation, chemosensitization, and EGF expression and promotes apoptosis. HIF-1α and HIF-2α blockades downregulate the expression of sex-determining region Y-box 2 (SOX2) and Krüppel-like factor 4 (KLF4), both of which are involved in the PI3K/AKT signaling pathway [67].

At the moment, Belzutifan, an oral HIF-2α inhibitor, is the only one approved by the FDA to treat VHL-associated renal cell carcinoma, hemangioblastomas, and pancreatic and neuroendocrine tumors [70]. Despite these results, there is insufficient evidence of long-term safety and efficacy when HIF inhibitors are combined with other therapies. Therefore, we proposed that the use of the pVHL, the main regulator of both HIF isoforms, could improve the current treatment due to the highly hypoxic microenvironment in GBM.

## 4. The Von Hippel–Lindau Protein (VHL), Suppressor of HIF Signaling

The Von Hippel–Lindau (VHL) tumor suppressor gene, located on chromosome 3p25~26, encodes a multifunctional protein known as the pVHL, which exists in four isoforms: pVHL-213, pVHL-160, pVHL-172, and pVHL-X1. Under physiological conditions, the pVHL is a stable protein with a half-life of approximately 24 h that functions as a critical regulator of all isoforms of HIFα subunit, by targeting them for degradation via the ubiquitin–proteasome pathway. The pVHL forms part of an E3 ubiquitin ligase complex by interacting with Elongin B, Elongin C, and Cullin-2, mediated by its BC-box motif. Under normoxic conditions, HIFα is hydroxylated by prolyl hydroxylase domain proteins (PHDs), allowing the pVHL to recognize and ubiquitinate HIFα, marking it for proteasomal degradation [71].

Loss of VHL function is associated with tumorigenesis, promoting angiogenesis, proliferation, and invasion due to uncontrolled HIF signaling [72]. Von Hippel–Lindau syndrome, an autosomal dominant hereditary disease, results from mutations that either inactivate the pVHL or lead to its abnormal production. This syndrome is characterized by multi-tumorigenic phenotypes due to HIF upregulation and increased expression of growth factors [73].

Germline mutations in VHL are associated with various diseases. For example, the biallelic mutation c.598C→T (R200W) and the synonymous variant p.V74V (c.222C→A) cause Chuvash polycythemia, an hereditary erythrocytosis. These mutations impair VHL function, which in consequence leads to chronic HIF stabilization, reduced mitochondrial respiratory capacity, and increased expression of genes such as BNIP3L (a mitophagy receptor) and MXI1 (a negative regulator of MYC involved in mitochondrial biogenesis) [74].

pVH is also regulated by the proteasome through Cullin2 and Cullin5, indicating its role within a tightly controlled feedback loop [75,76,77].

In addition to its canonical role in HIF regulation, the pVHL also interacts directly with p53, stabilizing and activating its transcriptional response following DNA damage. This interaction involves the recruitment of ATM kinase and histone acetyltransferases, leading to the transcription of p21, BAX, PUMA, and NOXA, which are involved in cell cycle arrest and apoptosis, thereby reinforcing its tumor-suppressive function [78,79].

In CNS tumors, Kanno et al. have shown that the presence of somatic mutation in the VHL gene and loss of heterozygosity on chromosome 3p are detectable in tumors, astrocytomas, oligoastrocytoma, and anaplastic astrocytoma [80]. Their findings suggest that the occurrence of hemangioblastomas and other glial tumors is associated with alterations in the VHL gene. Protein interaction network analysis has identified VHL as a core regulatory gene in GBM. These studies proposed that GBM cells express molecules that lead to VHL inactivation, contributing to HIF stabilization and promoting tumor growth [81].

### pVHL Regulation and Oncogenic Pathways That Favor Glioblastoma Growth

The pVHL plays a multifaceted role in GBM, acting as a critical tumor suppressor and regulator of hypoxia signaling. pVHL expression is reduced in GBM, suggesting that upstream regulatory mechanisms impair its function or stability. For example, Daam2 protein, a component of the WNT receptor complex, is overexpressed in GBM and inversely correlates with the pVHL. Daam2 directly interacts with the pVHL, promoting its ubiquitination and degradation, which results in proliferation and tumorigenesis [82] (Figure 2, left side).

Another pathway of pVHL regulation in glioma is through microRNAs (miRNAs). miR-23b, highly expressed in various GBM cell lines (U373, A172, LN229, U87, LN428, LN308) and in tumor tissue samples, binds to the 3′UTR of the VHL gene, inhibiting its expression and enhancing β-catenin/Tcf-4 activity and the HIF-1a/VEGF pathway and leading to enhanced invasion and tumor cell proliferation [83]. Similarly, miR-566 and miR-150 downregulate pVHL expression, promoting the HIF, VEGF, and matrix metalloproteinase (MMP-2 and MMP-9), thereby enhancing glioma proliferation, migration, invasiveness, glucose metabolism, and resistance to apoptosis [84,85] (Figure 2, left side).

The ubiquitin–proteasome system tightly regulates the pVHL. The E3 ligase FBXO22 is overexpressed in glioma and correlates with poor prognosis, apparently by promoting pVHL degradation [86]. In addition, molecular chaperones are essential for pVHL folding and stability. The TCP1 ring complex (TRiC), (a chaperonin complex), HSP70, and pre-folding VBP-1 contribute to pVHL folding and stabilization, while HSP90 targets the misfolded pVHL for degradation [87,88]. Interestingly, HSP70 overexpression has been associated with better prognosis, possibly by supporting pVHL stabilization and enhancing tumor cell recognition by NK cells following radiotherapy [89].

Additionally, murine double minute-2 protein (MDM2), another E3 ligase protein, is responsible for p53 (a tumor suppressor protein) ubiquitin degradation. It is overexpressed in around 14% of GBM cases, binds to pVHL under hypoxic conditions, and promotes neddylation, impairing the suppressive VHL-p53 complex [90,91]. Also, inhibitor of DNA binding 2 (ID2) is a protein mainly expressed in response to stress such as hypoxia; drives the adaptive cellular response to metabolic stress, suppressing ROS production from mitochondria, reducing mitochondrial damage, and promoting tumor cell survival during glucose deprivation; and has also been involved in GBM-associated pVHL suppression. Under hypoxia, ID2 directly binds and disrupts the VHL-Elongin C complex, stabilizing the HIF in tumor cells and promoting survival during metabolic stress [92] (Figure 2, left side).

A novel regulatory axis involved profilin-1 (Pfn-1) phosphorylation at Tyr129 GBM and presents high levels of phosphorylation of profilin-1 (Pfn-1) at Tyr129. Pfn-1 protein regulates actin polymerization and cytoskeletal growth, which augments microvascular density by binding to VHL and inhibiting VHL binding to HIF-1α (Figure 2, left side). Thus, inducing HIF-1α-mediated expression of angiogenic growth factors such as VEGF-A, basic fibroblast growth factor (bFGF), placenta growth factor (PlGF), and platelet-derived growth factor (PDGF) results in aberrant vascularization and tumor hypoxia, leading to GBM progression [93].

The tumor suppressor pVHL plays an essential role in cell cycle arrest by interacting with cyclin-dependent kinase inhibitors (CDKIs) such as p21, p27, and p57 (Figure 2, left side). These proteins are crucial negative regulators of cell proliferation. They are usually altered in GBM, leading to the constitutive activation of cyclin-dependent kinases (CDKs), a family of enzymes—serine/threonine kinases—responsible for controlling cell cycle progression and transcription regulation resulting in tumor proliferation. The pVHL binds to the N-terminal CDI domain of these inhibitors, prolonging their half-life. Disruption of this interaction—either through mutation in CDKIs or loss of pVHL—can impair cell cycle arrest, contributing to tumor growth. Notably, low expression levels of CDKIs and disrupted interaction with the pVHL have been correlated with poor prognosis in GBM patients [94,95].

Beyond cell cycle control, the pVHL modulates the nuclear transcription factor NF-kB, which is usually overactivated in GBM and is associated with growth, migration, and resistance to therapy. pVHL-mediated inhibition of NF-κB occurs through recruitment of casein kinase 2 (CK2) (Figure 2, left side). CK2 promotes phosphorylation of CARD 9, a known NF-κB agonist, in the C-terminal region. This phosphorylation impairs CARD9′s ability to activate NF-κB, thereby decreasing downstream signaling [96]. Since NF-κB is linked to the expression of the DNA repair enzyme MGMT, which confers resistance to chemotherapy, pVHL stabilization may restore therapeutic susceptibility [97].

The functional consequences of pVHL loss in GBM are broad and integrated into the oncogenic pathways. Among them, the pVHL indirectly regulates TGF-α via HIF degradation and promotes EGFR degradation via HIF-independent pathways [68].

Also, the TGF-β/TGF-β type I receptor (TBRI or ALK5) signaling pathways, which are a target of the pVHL that mediates ALK5-K48-linked poly-ubiquitination and drives its posterior proteasomal degradation [98] and disrupts TGF-β signaling. Additionally, the pVHL targets the SMAD2/3 proteins, a downstream effector of TGF-β, for ubiquitination by binding to the conserved LxLxxP motif in the C terminus of the MH2 domain [99] (Figure 2, left side).

Fibronectin (FN), an extracellular matrix (ECM) protein commonly overexpressed in GBM, contributes to tumor aggressiveness by promoting cell proliferation, migration, and angiogenesis. It involves the deposition and aberrant assembly of fibrils, which can function as a fibrillar scaffold to encourage the assembly of matrix proteins and confer resistance to ionizing radiation. Moreover, it facilitates ECM remodeling through actin cytoskeleton reorganization [100]. Interestingly, studies indicate that the pVHL can regulate FN at different levels: it positively controls FN mRNA levels in an HIF-independent manner and promotes FN deposition and proper assembly at the ECM, thereby influencing ECM composition and integrity [101] (Figure 2, left side). Furthermore, pVHL neddylation by NEDD8, a ubiquitin-like protein, is critical for ensuring proper ECM assembly and suppressing tumor progression [102].

In addition to its established roles in protein degradation and signaling modulation, the pVHL also influences epigenetic regulation in GBM. The loss or inactivation of VHL causes whole-genome changes in DNA methylation in different cell types [103,104], promoting the expression of *L2HGDH* and *D2HGDH* (2-hydroxyglutrate dehydrogenases), resulting in the increase in 2-hydroxyglutarate and specifically its L-isomer. Accumulation of 2-hydroxyglutarate results in a widespread hypermethylation phenotype independent of IDH mutations. Such epigenetic alterations can silence tumor suppressor genes, thereby facilitating glioma progression. In contrast, restoration of the functional pVHL has been shown to reverse this hypermethylation and decrease tumor volume [105].

Emerging evidence also describes the role of the pVHL in shaping the immune landscape of GBM. In VHL−/− tumors, the elevated expression of VEGF drives aberrant angiogenesis and contributes to immune evasion. Specifically, the VEGF/VEGFR2 pathway induces the expression of T cell exhaustion markers such as TOX on CD8+ T cells, rendering them less effective at targeting and killing tumor cells. This immunosuppressive microenvironment suggests a critical function of the pVHL in preventing antitumor immunity; by inhibiting the HIF and consequently reducing VEGF levels, the pVHL may indirectly favor the proper function of CD8+ T cells against tumor cells [106].

Several studies have demonstrated the immunosuppressive role of VEGF-A within the tumor microenvironment, particularly its contribution to T cell exhaustion, a key mechanism of immune evasion in cancer. In a seminal study, VEGF-A secreted by tumor cells was shown to directly induce the expression of inhibitory immune checkpoint receptors associated with CD8+ T cell exhaustion. When increasing concentrations of recombinant VEGF-A were added to in vitro T cell cultures, a dose-dependent upregulation of PD-1, Tim-3, CTLA-4, and Lag-3 was observed. These markers are typically associated with exhausted cytotoxic T lymphocytes. This immunosuppressive effect could be reversed through pharmacological inhibition of tyrosine kinase inhibitor (TKI) that blocks vascular endothelial growth factor receptors 1, 2, and 3 (VEGFR1, VEGFR2, and VEGFR3) in tumor models [107]. Supporting these findings, a preclinical study in small cell lung cancer (SCLC) showed that VEGF/VEGFR signaling promotes the expression of PD-1 and TIM-3 on tumor-associated T cells, contributing to an exhausted phenotype. The combined therapy using anti-VEGF and anti-PD-L1 antibodies was able to rescue T cell function [108].

## 5. Could the pVHL/HIF Axis Be a Real Target in GBM?

GBM is one of the most aggressive and treatment-resistant forms of brain cancer, characterized by hypoxia, aberrant vascularization, and intratumoral heterogeneity. As we have discussed among the regulatory pathways that drive GBM, the pVHL-HIF axis emerges as a central regulator. Under physiological conditions, the pVHL acts as a tumor suppressor by targeting the HIF-α subunit for proteasomal degradation, thereby limiting pro-angiogenic, metabolic, and proliferative responses. However, in GBM, the pVHL expression is downregulated or functionally inactivated by epigenetic repression, ubiquitin-mediated degradation, or disruption of the molecular chaperone system. This loss of function results in persistent HIF activation, enhancing the oncogenic and pro-angiogenic environment that promotes GBM progression and aggressiveness.

Experimental studies provide evidence that restoring pVHL expression produces antitumor effects. In U87, LN229, and C6 glioma cells, pVHL overexpression results in the reduction in HIF-1α, VEGF, and Bcl-2 as well as enhanced apoptosis, G0/G1 cell cycle arrest, reduced tumor growth, and invasion [72,83,108]. Importantly, pVHL restoration also downregulates glioma stem-like cells markers such as CD133, by inhibiting STAT3, JAK 2, and Elongin A, impairing self-renewal and tumorigenicity [109].

To address potential pVHL restoration strategies, the use of PROTACs that utilize VHL as an E3 ligase has proven to be a promising tool for cancer treatment; however, one of the main limitations is that they are large and highly polar molecules, and their bioavailability is low, which complicates oral administration and proper tissue and cell distribution. They also depend on VHL expression in tumor cells, which can affect their efficacy [110]. Another strategy for pVHL restoration involves using different vectors to increase pVHL expression in tumor cells. However, the challenge of identifying the correct vector that ensures efficient transfection has become a limitation for this approach.

## 6. Conclusions

The pVHL-HIF axis constitutes a central pathologic mechanism in GBM, linking hypoxic signaling to angiogenesis, metabolism, immune modulation, oncogenesis, and chemotherapy resistance. As discussed above, the pVHL directly regulates several molecules involved in cancer progression in an HIF-independent manner. Thus, therapeutic strategies aiming for restoration or mimicking pVHL function offer a promising approach for developing target therapies against GBM.

For this reason, we propose different approaches such as the use of PROTACS, viral transfection, and other delivery methods for restoring VHL level and function. Further development in the efficacy of such approaches will lead to a novel treatment for GBM patients.

## Data Availability

Not applicable.
